# Prognostic Significance of mTOR and PTEN in Patients with Esophageal Squamous Cell Carcinoma

**DOI:** 10.1155/2015/417210

**Published:** 2015-08-18

**Authors:** Jianjun Lu, You Pan, Xin Xia, Yong Gu, Yiyan Lei

**Affiliations:** Department of Thoracic Surgery, The First Affiliated Hospital, Sun Yat-sen University, Guangzhou 510080, China

## Abstract

The prognostic value of mTOR in ESCC is much controversial; this study aimed to determine the prognostic importance of mTOR and PTEN in patients with ESCC. A total of 148 consecutive patients who underwent esophagectomy from 2010 to 2012 were included in this study, tested by western bolt and immunohistochemistry for mTOR and PTEN expression. Correlation coefficient was calculated using Pearson's correlation test. The 3-year overall survival (OS) and disease-free survival (DFS) were calculated in relation to the two markers. 94 (63.5%) of 148 were mTOR high expression, and PTEN high expression was detected in 46 (31.1%) of the 148 patients with ESCC. The Pearson correlation coefficient revealed a significant negative correlation in two proteins (correlation coefficient = −0.189, *P* < 0.005). The 3-year OS and DFS time in the mTOR-high group was 23.9 and 18.4 months, respectively, and the time in the mTOR-low group was 33.9 months and 31.4 months, respectively. The difference of survival rate between the two groups remained statistically significant. mTOR-low or PTEN-high patients had better 3-year rates of OS and DFS than mTOR-high or PTEN-low group (*P* < 0.001 by the log-rank test). This study also found that mTOR was an independence prognostic factor by multivariate analysis.

## 1. Introduction

Esophageal cancer, one of the most common upper gastrointestinal tract malignant neoplasms, is the eighth most common cancer and the sixth leading cause of cancer-related mortality in the world [1, 2]. In China, esophageal cancer ranks the 5th most common diagnosed malignant tumors and 4th leading cause of cancer-related mortality. Esophageal cancer can be divided into two main pathological types: esophageal squamous cell carcinoma (ESCC) and esophageal adenocarcinoma. Esophageal adenocarcinoma is a tumor with high incidence rate in America and Europe; however, ESCC remains the most predominant form of esophageal cancer in China. Although the development of advanced therapeutic techniques has been made in the treatment of ESCC, including surgery, chemotherapy, radiation, or comprehensive treatment, the prognosis of ESCC patients is still poor, in which the overall 5-year survival rate of patient after surgery is only about 12% [3]. Owing to the lack of the effective method for early diagnosis, the ESCC patients are being diagnosed at late advanced disease stage and suffering dysphagia and low survival. Therefore, there is a great need for the pathogenesis of ESCC to disclose more biomarkers and provide clues for early screening and prevention.

Mammalian Target of Rapamycin (mTOR) is an atypical serine/threonine kinase that belongs to the phosphoinositide kinase-related family of protein kinases (PIKKs). mTOR assembles with several proteins to form two functionally and structurally multiprotein distinct complexes: mTOR complex 1 (mTORC1) and mTORC2 [4]. mTOR, as an essential integrator of growth factor-activated and nutrient-sensing pathways, plays a crucial role in various cellular processes, including protein, lipid and nucleotide synthesis, proliferation, differentiation, autophagy, apoptosis, and metabolism, via distinct signaling pathways [5, 6]. A series of previous clinical studies have demonstrated that mTOR is overexpressed and upmodulated in a wide variety of human tumors, such as lung cancer [7], breast cancer [8], hepatocellular cancer [9], and ovarian cancer [10]. Activation of mTOR, achieved through phosphorylation and overexpression in cell cycle regulation, inhibits cell apoptosis and accelerates cell proliferation which may lead to a tumorigenesis [11]. The mutation of phosphatase and tensin homolog (PTEN), the primary negative regulator of PI3K/Akt signaling, are detected in more than 70% of patients with the Cowden syndrome (CS), and these patients are at increased risk for breast, endometrial, thyroid, and renal carcinomas [12]. In most of sporadic cancers, mTOR activation is the result of activating mutation of PI3KCA [13], or deletion or loss-function of upstream regulator genes encoding TSC1/2 (tuberous sclerosis complex 1/2) [14], LKB1 (liver kinase B1) [15], or PTEN [16]. Since mTOR is involved in multiple aspects of tumorigenesis, while PTEN is a tumor suppressor, it is assumed that abnormal expression of these two kinds of protein affects patient prognosis and represents a novel target for therapy. The significance status of two proteins is predicting prognosis and survival time in a wide variety of tumor; however, there is an absence of data in the relationship between the proteins and the malignancy of ESCC. In this study, we hypothesized that the abnormal expression of mTOR and PTEN in resected ESCC would be associated with poor clinical outcomes.

## 2. Methods and Materials

### 2.1. ESCC Tissues Collection

In this study, 148 ESCC patients who underwent surgery in the Thoracic Department of The First Affiliated Hospital, Sun Yat-sen University, Guangzhou, China, from 2010.01 to 2012.12 were enrolled. Patients with prior malignancies, those with a second primary tumor, or those who received neoadjuvant chemotherapy and/or radiotherapy were excluded. The cases were selected consecutively on the basis of availability of resection tissue and follow-up data. Histological diagnosis was determined by pathologist according to the American Joint Commission on Cancer Staging (AJCC) criteria. Clinical data were reviewed retrospectively using written and electronic medical records.

### 2.2. Follow-Up

Patients accepted a telephone follow-up every three months until death or for at least 3 years. Follow-up time was calculated from the date of surgery to death or the date of the last contact. These data were used to analyze the relation between mTOR and PTEN expression level and tumor characteristics and clinical outcomes such as clinical stage, tumor size, lymph node status, nuclear grade, disease-free survival (DFS) rate, and overall survival (OS) rate.

### 2.3. Western Blot Analysis

For western blot analysis of mTOR or PTEN, ESCC tissue (60 mg) from patient was homogenized in 3 mL ice-cold homogenization buffer (containing protease inhibitor, calcineurin inhibitors, and PMSF). After centrifugation (12,000 ×g, 10 min at 4°C), the supernatant was collected and protein concentrations were determined by using a bovine serum albumin (BSA) standard line. Equal amounts of proteins were separated on sodium dodecyl sulfate polyacrylamide gel (SDS-PAGE) and then transferred electrophoretically onto polyvinylidene difluoride (PVDF) membranes (Millipore, Bedford, MA, USA). The blotted membranes were blocked with 5% nonfat dry milk (sigma) in Tris-buffered saline with 0.1% Tween-20 (TBST) and then incubated at 4°C overnight with polyclonal antibodies against mTOR or PTEN (abcam), respectively. After six rinses with TBST at 5-min intervals, the membranes were incubated for 45 min with horseradish peroxidase-labeled goat anti-rabbit IgG. Immunoblotting with anti-GAPDH antibody was used as an internal control to confirm equivalent protein loading. The membranes were exposed to Odyssey CLx Imager and image capture and data analysis were done with Odyssey Software (LI-COR Biosciences, America).

### 2.4. Immunohistochemistry (IHC)

Expression of the mTOR or PTEN protein in tissue sections was measured by immunohistochemistry staining. The sections were heated at 65°C for 2 hours, deparaffinized in xylene, and rehydrated in a graded series of alcohol solutions. These sections were covered with 10 mM sodium citrate buffer (pH 6.0), heated in a pressure cooker for 5 minutes, and treated with 1%(v/v) normal goat serum for 15 minutes. The mTOR antibody (2983S, Cell Signal Technology, Co. Ltd., USA) or the PTEN antibody (9559S, Cell Signal Technology, Co. Ltd., USA) had been produced and its reliability had been confirmed by western blotting. The antibody was incubated with the sections at 4°C overnight in a humid chamber, at a 1 : 100 dilution. The antibody complex was detected by incubation with an avidin-biotin-peroxidase complex solution and visualized by 3,3-diamino-benzidine. The sections were counterstained with hematoxylin for 2 min. Normal esophageal tissue, serving as the negative controls, and esophageal cancer tissue (known to express mTOR) serving as positive controls were processed in the same way. However, normal esophageal tissue serves as the positive controls, and esophageal cancer tissue serves as negative controls for PTEN.

### 2.5. Evaluation of IHC Staining

All slides were evaluated independently by two pathologists blind to patients and their clinical information. Positive staining of the cytoplasm was evaluated in at least five fields in 400x magnification. mTOR and PTEN IHC were initially scored into four groups according to the extent and intensity of cytoplasmic staining of the tumor cells, respectively. Staining extent was scored according to the percentage of positive staining tumor cells seen. Staining extent was scored as negative (<5%) [0]; weak (5–25%) [1]; moderate (25–50%) [2]; or strong (>50%) [3]. Staining intensity was scored according to the degree of positive staining tumor cells seen. Staining intensity was marked as nongranulated [0]; low grade (light yellow) [1]; moderate (brownish yellow) [2]; or strong (reddish brown) [3]. Total score was equal to staining extent multiplied by staining intensity. The specimens whose overall scores were 4 to 9 were defined as high-density protein, which were considered positive overexpression. Low expression group included negative expression or low expression samples with overall scores of 0 to 3.

### 2.6. Statistical Analysis

All statistical analysis was performed using the SPSS software (SPSS version 19.0; IBM SPSS Inc., Chicago, IL, USA). The correlation of mTOR and PTEN protein expression with clinic pathologic characteristic of ESCC patients was assessed by the chi-square test. According to mTOR status or PTEN status patients included in our study were divided into two groups. The Pearson correlation test was used to analyze the correlation between mTOR and PTEN expression by IHC. Kaplan-Meier curves were used to model DFS (i.e., the time from day of surgery to recurrence or death) and OS (i.e., the time from day of surgery to death) and the log-rank test was used to compare differences in survival between groups. Univariate and multivariate analyses of the prognostic factors were examined by Cox's proportional hazard model. All the statistical tests were two-sided, and a *P* value <0.05 was considered significant.

### 2.7. Ethical Approval

Studies using human tissue were reviewed and approved by the Committees for Ethical Review of Research involving Human Subjects of Sun Yat-sen University (Guangzhou, China). All procedures complied with the ethical guidelines and all participants provided signed informed consent.

## 3. Results

### 3.1. mTOR Expression in ESCC and Normal Esophageal Tissues

**Figure 1:**
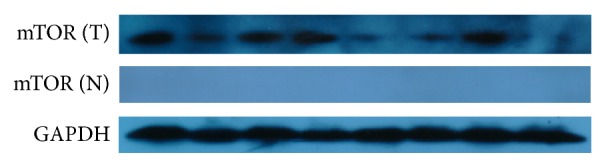
Western blot analysis of mTOR expression in ESCC tumor tissues (ESCC: esophageal squamous cell carcinoma).

**Figure 2:**
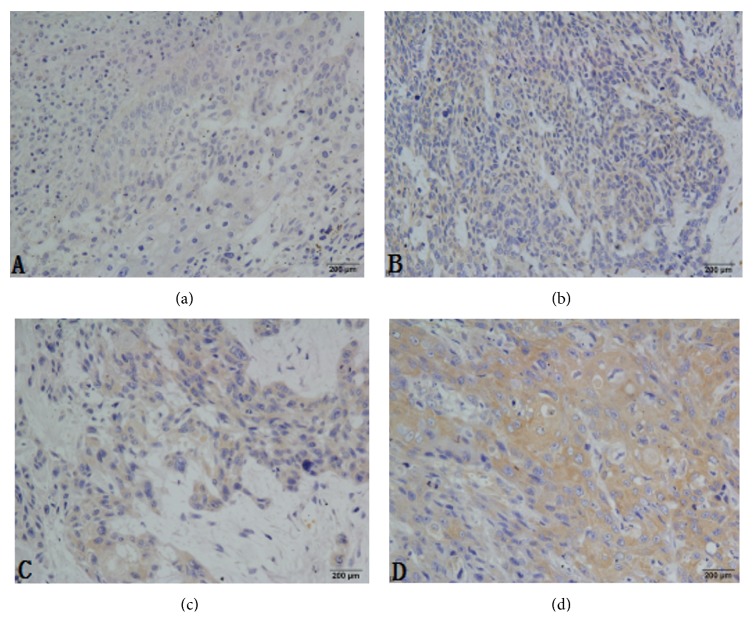
Immunohistochemistry (IHC) analysis of mTOR expression in normal and ESCC tissues. (a) Normal esophageal tissue showed nearly negative expression of mTOR (400x); (b) low expression of mTOR was shown in ESCC patient samples (400x); (c) moderate expression of mTOR was detected in ESCC samples (400x); (d) high expression of mTOR was detected in ESCC samples (400x). ESCC: esophageal squamous cell carcinoma.

The mTOR was detected by western blot and IHC in this study. In the ESCC tumor samples, mTOR protein was elevated by western blot analysis (Figure 1). A total of 148 groups of ESCC tumor sections and 10 tumor-adjacent normal esophageal tissue sections were detected by IHC (Figure 2). In the cohort of 148 ESCC patients, high expression of mTOR was detected in 94 of 148 (63.5%) patients and low expression of mTOR was detected in 54 of 148 (34.5%) patients, including negative expression of mTOR which was detected in 21 of 54 (38.9%). The overall scores of all normal esophageal sections were below 2.

### 3.2. PTEN Expression in ESCC and Esophageal Tissues

**Figure 3:**
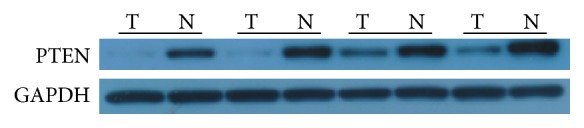
Western blot analysis of PTEN expression in ESCC tumor and normal adjacent tissues (ESCC: esophageal squamous cell carcinoma).

**Figure 4:**
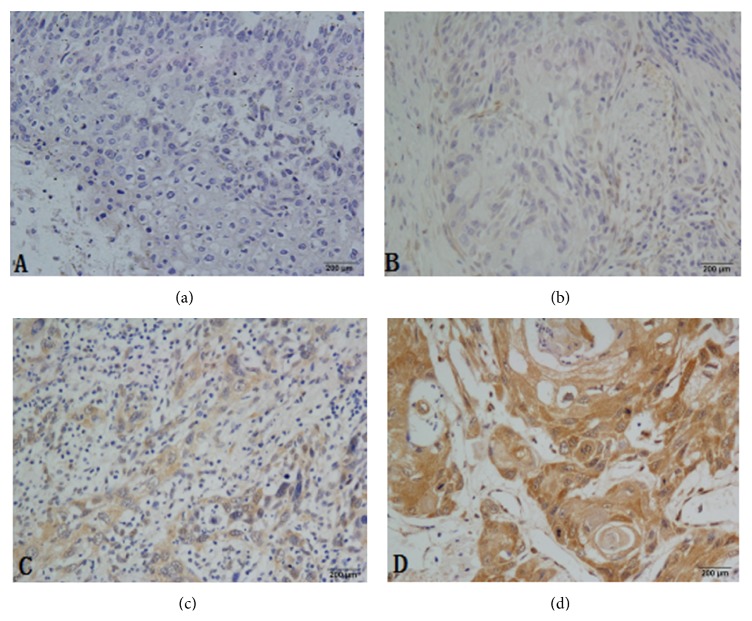
Immunohistochemistry (IHC) analysis of PTEN expression in normal and ESCC tissues. (a) Nearly negative expression of PTEN was shown in ESCC patient samples (400x); (b) low expression of PTEN was shown in ESCC patient samples (400x); (c) moderate expression of PTEN was detected in ESCC samples (400x); (d) high expression of PTEN was detected in normal esophageal samples (400x). ESCC: esophageal squamous cell carcinoma.

The PTEN protein was also detected by western blot and IHC. PTEN protein was elevated compared with the normal adjacent tissues by western blot analysis (Figure 3). In this study, high expression of PTEN was detected in 46 of 148 (31.1%) patients and low expression of PTEN was detected in 102 of 148 (68.9%) patients. The overall scores of all normal esophageal sections by IHC were positive expression (Figure 4).

### 3.3. The Relationship between mTOR Expression and Pathological Characteristics in the ESCC Patients

In this study, clinical characteristics of the 148 ESCC patients, including gender, age, tumor differentiation, pathological stage, tumor status, and lymph node status, are summarized in Table 1. 114 males (77%) and 34 females (23%) are included, and the age of the patients ranged from 39 to 84 years (median of 59 years). The pathologic stages (p-stages) of patients range from I to III which are evaluated and determined according to the criteria of the AJCC. The performance status (PS) of all patients is 0. The follow-up period ranged from 3 to 36 months, with a median of 27 months. The high expression of mTOR protein was closely related to the pathological stage (<0.001); the overexpression staining was detected in 6 of 20 in the p-stage I patients, 16 of 46 in p-stage II patients, and 72 of 82 in p-stage IIIA patients (Table 1). No significant association was found between mTOR overexpression and other clinic pathologic features such as tumor length, gender, and age in our study (Table 1).

### 3.4. Analysis of mTOR and PTEN

94 of 148 patients were mTOR-high expression. 54 of 148 mTOR-low expression tumors were stained positive for PTEN by in situ hybridization, but 23 of 54 mTOR-low tumors were stained over positive for PTEN. The presence of mTOR and PTEN expression in tumors had significant differences (*P* < 0.05). mTOR expression was strongly negative associated with PTEN expression (correlation coefficient = −0.189) (Table 2).

### 3.5. Survival Analysis of PTEN and mTOR

**Figure 5:**
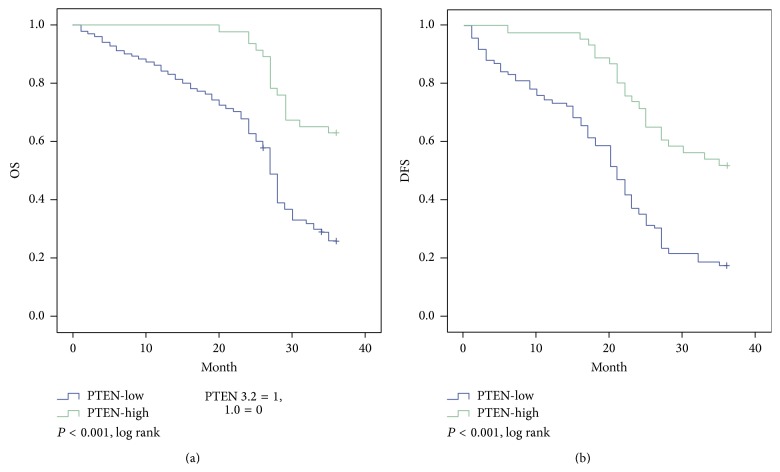
Kaplan-Meier estimates of survival among the study patients, according to tumor PTEN expression. For 3-year overall survival time (a) and 3-year progression-free survival time (b), PTEN expression was significantly associated with improved outcomes (*P* < 0.001; *P* < 0.001, resp.).

**Figure 6:**
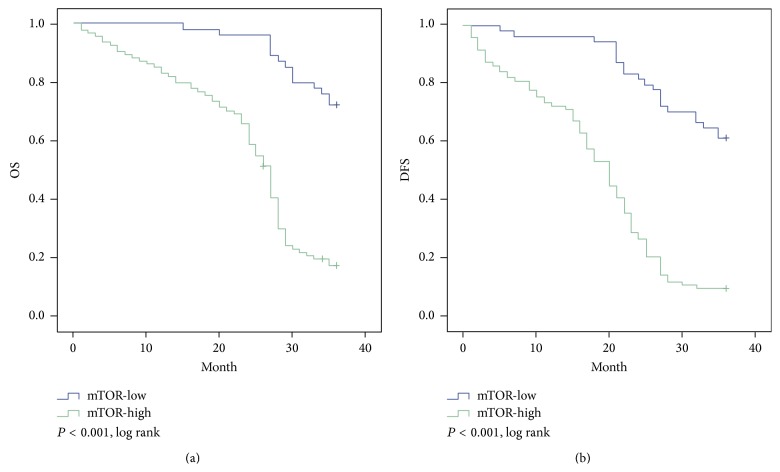
Kaplan-Meier survival analysis of mTOR expression in total cohort of ESCC patients (*n* = 148); (a) high expression of mTOR was closely correlated with inferior overall survival; (b) disease-free survival in ESCC patients. The median survival time for patients with high and low expression of mTOR was 23.9 versus 33.9 months for OS (*P* < 0.001) and 18.4 versus 31.4 months for DFS (*P* < 0.001) (ESCC: esophageal squamous cell carcinoma).

Based on Kaplan-Meier analysis, OS was superior in the PTEN-high expression group compared with that in the PTEN-low group, with 3-year survival time of 32.8 months and 25.2 months, respectively (*P* < 0.001; Figure 5(a)). PTEN-high patients also had statistically significantly better DFS than PTEN-low patients; the 3-year DFS were 29.7 months and 20.2 months, respectively (*P* < 0.001; Figure 5(b)). Using mTOR status as a stratification factor, the survival results were inversely correlated with the results based on PTEN expression. OS was superior in the mTOR-low group compared with that in the mTOR-high group, with 3-year survival time (33.9 months and 23.9 months, resp.; *P* < 0.001, Figure 6(a)). The low group also has longer DFS compared with high group. The 3-year DFS were 31.4 months and 18.4 months, respectively (*P* < 0.001, Figure 6(b)).

Cox regression analysis of OS and DFS, including prognostic factors of T category, N category, p-stage, differentiation, gender, age, tumor length, and mTOR status, suggested that T category, N category, p-stage, differentiation, and mTOR status were the significant factors in multivariable analysis (Table 3) for OS and only T category, N category, and mTOR status were the significant factor in multivariable analysis for DFS (Table 4). Furthermore, mTOR was an independent prognostic factor for DFS (hazard ratio, 2.229; 95% CI, 1.123–4.425; *P* = 0.022, Table 2) and OS (hazard ratio, 2.033; 95% CI, 1.117–3.701; *P* = 0.020, Table 3) in the cohort of 148 resected ESCC patients.

## 4. Discussion

As one of the high-occurrence upper gastrointestinal tract carcinomas in our country, ESCC had been characterized by high invasive, aggressively malignant, poor prognosis, and the most prevalent of all forms of gastric cancers and the five-year survival rate was only 20 percent. It was short of effective early diagnostic methods and powerful treatment for this cancer. However, as the technology improves, it may help us find out possible therapeutic targets and identification of new diagnostic and prognostic markers. Further, intensive study on genetic characterization will help to understand the occurrence and development of cancer and facilitate the development of new personalized therapeutic strategies. Some research indicates that mTOR expression can be increased in most human cancers and growing evidences demonstrate that mTOR plays a role not only in the formation of tumors, but also in the cancer progression [17–19].

Our study initially focused on description of the mTOR expression of ESCC by the western blot and IHC; the western blot results matched well with the data of IHC and revealed that there was variability of mTOR expression in the ESCC. It was likely to provide potential way to distinguish ESCC tissues and normal tissues. Our data suggested that the mTOR-high expression was a universal phenomenon in ESCC and may play a potential role in its development. Li et al. found that mTOR expression was higher in ESCC tissues as the same with our study [20], and Hou et al. also observed it would increase the sensitivity of the EC9706 cells to cisplatin at proliferation in vitro and in vivo by mTOR siRNA [21]. What is more, our results suggested that there was a consistent trend that mTOR overexpression rate was associated with the status of lymph node metastasis, the pathological differentiation, and the rise of clinical stage. Through the further analysis of the relationship of mTOR differential expression between the OS and DFS, we found that the OS and DFS of patients with mTOR-high expression group are significantly lower than the counterpart with mTOR-low expression group (OS: 23.9 months versus 33.9 months, *P* < 0.001; DFS: 18.4 months versus 31.4 months, *P* < 0.001, resp.), which was similar with Hirashima's results, that found p-mTOR-positive patients experienced high mortality [22].

Considering the intricacy of tumor formation, it is impossible that the mTOR is the only one ascribed to the ESCC. Deep explorations revealed that mTOR can interact with a broad spectrum of other biological molecules, such as AKT and PI3K, promoting the cancer cells growth [23]. But it is not clear whether it could play a role by inhibiting the expression of tumor suppressor gene, so we also investigate the expression of PTEN in the ESCC.

PTEN, as a tumor suppressor, is an important marker of good prognosis in various neoplasms [24]. Some of the existing studies have demonstrated its value as a marker of a good prognosis in breast and prostate cancer [25, 26], but much remains to be determined concerning the function of PTEN in tumor initiation and cancer progression, including digestive system neoplasms. In this study, we identified the differentially expressed PTEN amongst the ESCC patients. Our research demonstrated that PTEN is a weekly expression in ESCC patients. However, the high expression of PTEN in ESCC patients is significantly different from one with lower expression in the OS and DFS; the patients with PTEN increased expression have more OS and DFS (OS: 32.8 versus 25.2, *P* < 0.001; DFS: 29.7 versus 20.2, *P* < 0.001, resp.), which means that the restraint of PTEN may prompt recurrence and poor prognosis in ESCC.

In order to define the relationship between mTOR and PTEN with respect to the occurrence and development and prognosis in the ESCC, we compare their expression in the ESCC. It is suggested that the mTOR positive patients have a universal phenomenon; however, the PTEN are lower expression. There is a significant difference between mTOR and PTEN; mTOR expression was strongly negative associated with PTEN expression (correlation coefficient = −0.189, *P* < 0.05).

Our present analyses show that mTOR and PTEN expression are associated with the prognosis of ESCC patients, while these are still some notable limitations. On one hand, the current study was based on relatively small samples; we need a multicenter study in larger population of ESCC and collect more samples to further verify the results. On the other hand, there is only 3-year follow-up in this study, which prompted us to add more follow-up times, making our argument more persuasive. Further limitations that stem from mTOR may serve as a novel molecular target to optimize individual therapy; we need further researches to be conducted to identify the precise signaling pathway of mTOR involved in the pathogenesis of ESCC.

## 5. Conclusion

This study demonstrates that mTOR expressed rate is high, while the positive expression rate of PTEN is low in ESCC tissues, and there is negative correlation between their expressions. The activation of mTOR or the suppression of PTEN may provide some conditions for ESCC to thrive. More work will be needed to determine whether the mTOR or PTEN can serve as new therapeutic targets and diagnostic biomarkers in ESCC.

## Figures and Tables

**Table 1:** Baseline characteristics of the study patients and their tumors, according to mTOR expression.

Characteristics	Total	mTOR-high	mTOR-low	*P* value
	*N* = 148	*N* = 94	*N* = 54	
Gender				0.686
Male	114	71	43	
Female	34	23	11	
Age				0.473
<65	51	30	21	
⩾65	97	64	33	
Tumor length				0.302
<5	64	44	20	
⩾5	84	50	34	
pT status				<0.001
pT1	21	6	15	
pT2	37	11	26	
pT3	76	64	12	
pT4	14	13	1	
pN status				<0.001
pN0	56	19	37	
PN1	92	75	17	
TNM stage (AJCC)				<0.001
I	20	6	14	
II	46	16	30	
III	82	72	10	
Differentiation grade				0.007
Well	20	13	7	
Moderate	81	43	38	
Poor	47	38	9	

AJCC: American Joint Commission on Cancer Staging; pT: pathological tumor stage; pN: pathological node stage.

**Table 2:** Correlation between mTOR and PTEN in the esophageal squamous cell carcinoma.

PTEN status	Total	mTOR-low	mTOR-high	Correlation coefficient
	*N* = 148	*N* = 54	*N* = 94	
Low	102	31	71	−0.189^*∗*^
High	46	23	23	

^*∗*^ *P* < 0.05.

**Table 3:** Multivariate analysis of overall survival.

Factors	Levels	Beta	*P* value	HR	95% CI
Gender	Male versus female	−0.107	0.679	0.898	0.54 to 1.493
Tumor length	⩾5 versus <5	−0.038	0.870	0.963	0.614 to 1.51
Age	⩾65 versus <65	0.274	2.610	1.316	0.815 to 2.124
T category	T1 + T2 versus T3 + T4	1.406	0.006	4.081	1.489 to 11.183
N category	N0 versus N1	2.930	0.000	18.728	4.084 to 85.878
p-stage	I versus II + III	−1.771	0.047	0.170	0.03 to 0.978
Differentiation	Well versus moderate + poor	1.756	0.001	5.790	2.033 to 16.491
mTOR	High versus low	0.710	0.020	2.033	1.117 to 3.701

HR: hazard ratio; CI: confidence interval.

**Table 4:** Multivariate analysis of disease-free survival.

Factors	Levels	Beta	*P* value	HR	95% CI
Gender	Male versus female	0.239	0.377	1.224	0.781 to 1.919
Tumor length	⩾5 versus <5	−0.144	0.502	0.866	0.569 to 1.318
Age	⩾65 versus <65	0.084	0.699	1.088	0.709 to 1.669
T category	T1 + T2 versus T3 + T4	1.643	0.001	5.170	1.956 to 13.664
N category	N0 versus N1	1.757	0.000	5.793	2.318 to 14.478
p-stage	I versus II + III	−2.79	0.621	0.757	0.251 to 2.286
Differentiation	Well versus moderate + poor	0.493	0.195	1.638	0.776 to 3.455
mTOR	High versus low	0.801	0.022	2.229	1.123 to 4.425

HR: hazard ratio; CI: confidence interval.
